# Foam Cushing: endocrine disruption from excessive hygiene zeal

**DOI:** 10.1007/s40618-026-02840-4

**Published:** 2026-02-20

**Authors:** Lucrezia Rossi, Valentino Marino Picciola, Alberto Gobbo, Guido Di Dalmazi, Matteo Magagnoli, Flaminia Fanelli, Maria Rosaria Ambrosio, Maria Chiara Zatelli

**Affiliations:** 1 Section of Endocrinology, Geriatrics and Internal Medicine, Department of Medical Sciences (MUR Excellence Department), University of Ferrara, Ferrara, 44124 Italy; 2 Division of Endocrinology and Diabetes Prevention and Care, IRCCS Azienda Ospedaliero-Universitaria di Bologna, Bologna, Italy; 3 Department of Medical and Surgical Sciences (DIMEC), Alma Mater Studiorum University of Bologna, Bologna, Italy; 4 Center for Applied Biomedical Research, Department of Medical and Surgical Sciences, Alma Mater Studiorum University of Bologna, Bologna, Italy; 5 Endocrine Unit, University Hospital S. Anna, Ferrara, 44124 Italy

**Keywords:** Endocrine disruptor, Exogenous Cushing syndrome, Glucocorticoid excess, Piperonyl butoxide, Anti-lice product, Adrenal Insufficiency

## Abstract

**Purpose:**

Cushing’s syndrome (CS) affects children in 10% of cases and is occasionally associated with the abuse of health-related products causing exogenous glucocorticoid (GC) excess. A 15-year-old girl presented with suspected CS: endogenous hypercortisolism and exposure to GC containing drugs were ruled out. She reported using an anti-lice foam for one year. After discontinuation, her clinical condition gradually and completely reversed. This study aims to investigate whether compounds, such as pyrethrins and piperonyl butoxide (PBO), contained in the anti-lice product have GC-like activity.

**Methods:**

Serum levels of pyrethrins and PBO in the patient and controls were measured by LC-MS/MS. To test whether PBO has GC-like effects, the GloResponse 9XGAL4UAS-luc2P HEK293 (GloHEK) cell line was incubated with PBO and luminescence (LUM) was measured.

**Results:**

During anti-lice product exposure patient’s PBO serum levels were ~ 25-fold higher than control samples. Six months after product discontinuation, PBO levels in patient’s serum decreased to levels comparable to controls. PBO did not affect the viability of GloHEK cells at the concentration employed in the luciferase assay (10 µM). Baseline GloHEK cell LUM was significantly increased by PBO (> 7-fold; *p* < 0.01), an effect reversed by a GC antagonist.

**Conclusion:**

PBO contained in the anti-lice product has a GC-like transcriptional activity. These results support the hypothesis that CS signs and symptoms were related to anti-lice product exposure, causing a foam-induced Cushing. The use of pharmaceutical and non-pharmaceutical products should always be investigated in patients with CS.

## Introduction

Cushing’s syndrome (CS) is a clinical condition caused by glucocorticoid (GC) excess, due to abnormal endogenous cortisol production or exogenous GC exposure [1, 2]. Central obesity, “buffalo hump” and rounded face characterize CS. In addition, facial plethora, easy bruising, thin skin, striae rubrae, myopathy and muscle weakness can be observed [3]. High GC levels cause hyperglycemia, abnormal protein catabolism, immunosuppression, neurocognitive changes, bone fragility and mood disorders [4]. Exogenous and endogenous GC excess share similar signs and symptoms. However, exposure to exogenous GC is generally associated with a more rapid onset of CS clinical signs as compared to endogenous hypercortisolism [3]. The use and misuse of oral, inhaled, injected, or topical drugs containing GC is the most common cause of exogenous CS [2]. In addition, over-the-counter herbal products, tonics, bleaching creams, and supplements may contain GC. Therefore, it is crucial to carefully investigate the use of these products during clinical evaluation of patients with suspected CS [1, 5, 6].

## Case report

A 15-year-old girl presented to our outpatient clinic with hirsutism, acne, oligomenorrhea, severe asthenia and significant weight gain over the last 3 months. Menarche occurred at 10 years of age with normal menstrual cycles; at diagnosis, she showed complete pubertal development (Tanner Scale P5G5) and mild hirsutism (Ferriman-Gallwey score = 12). Intense physical activity and medications were denied (including topical steroids, inhaled GCs, supplements, phytotherapics). Body weight and height were 68 kg (90th -97th percentile) and 164.5 cm (75th -90th percentile), respectively. At presentation the target height (158.5 ± 8 cm) had already been reached and blood pressure was 90/60 mmHg. Striae rubrae on the hips and arms were observed. Hormonal tests showed low basal ACTH and cortisol plasma levels with low 24-hour urinary cortisol levels. The remaining assessments are reported in Table 1. A 250 µg ACTH test showed an impaired response of plasma cortisol levels, suggesting a picture of adrenal insufficiency, supporting the hypothesis of chronic exogenous GC exposure. Replacement therapy with cortisone acetate (25 mg daily split in two administrations) was started, with improvement in asthenia. After inquiring the parents for more accurate personal history, including exposure to endocrine disruptors, we found that the girl had been exposed to an anti-lice foam treatment containing natural pyrethrins for approximately one year, with an increased frequency in the last three months (almost daily). Application of this product was discontinued immediately. After six months plasma cortisol (167 ng/ml) and ACTH (32.2 pg/ml) levels went back to normal, as well as adrenal androgen levels. Clinically, the patient reported regular menstrual cycles, weight loss, and marked improvement in hirsutism, acne, and striae rubrae. Cortisone acetate therapy was stopped, and the patient was found healthy at the subsequent follow-up.

**Table 1:** Patient’s biochemical workup at first observation

Urinary free cortisol (µg/die)	Results	Reference range
	25	58–503
Serum cortisol (ng/ml)	7	67–226
ACTH (pg/ml)	4.6	0–46
Dehydroepiandrosterone sulfate (µg/dl)	26	10–60
Testosterone (ng/ml)	< 0.1	10-0.75
Δ^4^-Androstenedione (ng/ml)	0.7	0.4–3.4
Progesterone (ng/ml)	< 0.049	0.31–1.52
17-hydroxyprogesterone (ng/ml)	0.317	0.2–1.3
FSH (mU/ml)	4.4	3.9–8.8
LH (mU/ml)	3.3	2.1–10.9
17-β-Estradiol (pg/ml)	73	22–115
PRL (ng/ml)	11.2	3.3–26.7
IGF-1 (ng/ml)	345.3	188–510
TSH (µU/ml)	1.18	0.25–4.5
FT4 (pg/ml)	8.7	5.5–12
Sodium (mmol/l)	137	136–145
Potassium (mmol/l)	4.3	3.5–5.3
Glucose (mg/dl)	88	70–110

All the reported analytes were assessed by standard methods available in our Hospital laboratory

This study investigates whether the anti-lice product contains compounds with GC-like activity, supporting the hypothesis that chronic exogenous GC exposure causes the CS picture in our patient. Anti-lice products are widely used in the pediatric population to treat and/or prevent head lice infestation [7].

## Materials and methods

### Subjects

The patient’s parents agreed to publish personal data and clinical history. Controls sample was represented by a pool of female serum leftover samples from routine analyses.

### Compounds

The employed anti-lice foam product contains pyrethrins and piperonyl butoxide (PBO). Pyrethrins are used as insecticides to treat parasitic infections such as scabies and lice; PBO is an insecticide and acaricide used to increase pyrethrin potency [8]. A stock mixture including pyrethrin I and II at about ~ 80 mg/ml concentration (PY-T-50, lot number: 0520-506-0723) was provided by Botanical Resources Australia (Ulverstone, Tasmania, Australia). PBO stock solution (lot number: R2006013) at 1 g/ml was provided by Endura S.p.A (Bologna, Italy). Dexamethasone (Dex) and Mifepristone (Mif) were purchased from Sigma-Aldrich (Milano, Italy). For cell culture experiments, Dex was dissolved in ethanol while Mif and PBO were dissolved in methanol as stock standard solutions (10 mM); further dilutions were prepared daily. Dex and Mif standard solutions were stored at 4 °C. Standard solutions were diluted in a charcoal dextran-stripped culture medium to obtain the final concentration. Solvent final concentration in the culture medium was 0.1% (both for ethanol and methanol) at the highest compound concentration; vehicle controls contained the highest solvent concentration.

### Pyrethrins and PBO measurement in serum

Two LC-MS/MS methods were developed on the Serie 200 modular HPLC (Perkin Elmer, Waltham, MA, USA) coupled with the API 4000 Qtrap (Sciex, Framingham, MA, USA) platform. Stock solutions were diluted at 0.5–1 µg/ml in methanol and kept at -20 °C. Compounds were infused at 10 µl/min in the electrospray Turbo Ion source to optimize two multiple reaction monitoring transitions for each compound. Optimized source parameters were temperature, 550 °C; ion spray, 5500 V; curtain gas, 15 psi; gas 1, 40 psi; gas 2, 40 psi. Compound dependent parameters are reported in Table 2.

**Table 2:** Analyte-dependent LC-MS/MS detection and quantitation parameters

Analyte	Molecular weight (g/mol)	Retention time (min)	Q_1_/Q_3_ Quantitative MRM	Q_1_/Q_3_ Qualitative MRM	DP (V)	CE (V)	CXP (V)	Measurement range (pg/ml)	Limit of quantitation in samples (pg/ml)
Pyrethrin I	328.4	6.28	329.3/161.0	329.3/132.9	50	12	10	22.8–5550	1.14
Pyrethrin II	372.4	4.76	373.4/161.0	373.4/132.9	90	15	10	22.8–5550	1.14
Piperonyl butoxide	338.4	11.57	356.4/177.0	356.4/119.0	60	18	10	9.8–1.25	1.96

Both LC methods were developed on the Luna Eclipse XDB-C18 3.5 μm 3 × 100 mm column, equipped with C18 4 × 2 mm guard column, operated at 500 µl/min flow and 45 °C oven temperature. For the pyrethrin panel, mobile phase A was 0.1 mM ammonium fluoride, whereas mobile phase B was methanol. The gradient started with mobile phase B at 40% from 0 to 0.5 min, increasing to 75% at min 1, and to 100% at min 7. The system was washed until min 8, then re-equilibration to 40% B occurred from min 9 to 12. For PBO method mobile phase A was 0.1 mM ammonium acetate and mobile phase B was methanol. The gradient started with mobile phase B% at 30% from 0 to 0.5 min, increasing to 65% at min 1, and to 85% at min 15. The system was washed with 100% B from min 15.1 to 16; afterward, the system was re-equilibrated to 30% until min 18. Retention times are reported in Table 2.

The day of the assay, calibrators were prepared in methanol to produce measurement ranges reported in Table 2. Sample extraction was performed by adding 2 ml of N-hexane: ethyl acetate mixture 9:1 to 500 µl of each serum. After vortexing 5 min and centrifuging 5 min at 3000 g, the organic phase was dried under gentle nitrogen flow. For pyrethrin analysis, samples were reconstituted with 25 µl methanol, 10 µl of which were injected in the LC-MS/MS system. For PBO measurement, extracts were reconstituted with 100 µl methanol, 20 µl of which were injected in the LC-MS/MS system. For both, autosampler was kept at 18 °C. The limit of quantitation (LOQ) in samples was 1.14 pg/ml for pyrethrin I and II, and 1.96 pg/ml for PBO (Table 2).

### Cell culture and cell number evaluation

The GloResponse 9XGAL4UAS-luc2P HEK293 cell line (Promega, Madison, WI, USA; RRID: CVCL_LB59) was generated by clonal selection of human embryonic kidney 293 (HEK293) cells, stably transfected with the pGL4.35[luc2P/9XGAL4UAS/Hygro] vector (RRID: NCBITaxon_659256), indicated from here on as GloHEK cells. Cells were cultured in DMEM high glucose (Invitrogen, Waltham, MA, USA), 10% defined fetal bovine serum (FBS) (Hyclone, Logan, UT, USA) and 200 µg/ml hygromycin B (Invitrogen). GloHEK cells were maintained at 37 °C in a humidified atmosphere with 5% CO_2_. Charcoal-stripped FBS and phenol-red-free medium were used in all cell viability and luciferase experiments. To evaluate the effects of PBO, Dex and Mif on viable GloHEK cell number we plated 10^5^ cells/well in 24-well plates. Cells were then treated with increasing concentrations (up to 100 mM) of each compound for 48 and 72 h. Control cells were treated with vehicle alone (1% ethanol). Cell number was evaluated by counting viable cells with a Bürker chamber. Results are expressed as percent mean viable cell number ± standard error (SE) vs. control cells from at least 4 replicates in 4 independent experiments.

### Transfection and Dual Glo luciferase assay

GloHEK cells were seeded in 96-well culture plates (Corning, Turin, Italy) at 10^4^ cells/well, in DMEM without phenol red (Invitrogen) containing 5% charcoal/dextran-treated FBS (Hyclone). The next day the cells were transfected with 100 ng pBIND-GR Vector (Promega) containing the GR ligand-binding domain, as previously described [5]. The day after, the cells were treated with 10 µM Dex, according to the manufacturer’s instruction, to verify whether transfection was effective. Then cells were treated with 10 µM Mif or PBO alone and in combination. Control cells were treated with vehicle solution. After 24 h, cells were assessed for Firefly and Renilla luciferase activity using the Dual Glo luciferase reporter assay (Promega) according to the manufacturer’s instructions. Luminescence (LUM) was measured by EnVision Multilabel Reader (PerkinElmer, Waltham, MA). Results are expressed as percent mean value ± SE relative light units (RLU) vs. control cells from 7 independent experiments in 3 replicates.

### Statistical analysis

The statistical analysis was performed using the t-test for two independent samples to assess individual differences between means. For multiple comparisons to assess differences between groups ANOVA test and Tukey post-hoc tests were performed. P-value < 0.05 was considered significant.

## Results

### Evaluation of serum and plasma pyrethrins and PBO levels

Pyrethrins and PBO concentrations were measured in sera from the patient and controls. Pyrethrin levels were undetectable in all samples (data not shown) and therefore were not further investigated. PBO levels in control samples were below the limit of quantitation at 1.96 pg/ml. At variance, levels in patient’s serum during anti-lice product exposure were ~ 25-fold higher as compared to control samples, independently of treatment with cortisone acetate. Six months after anti-lice product withdrawal, PBO levels in patient’s serum were undetectable, similarly to those detected in control samples (Table 3).

**Table 3:** PBO concentrations in serum samples

Sample	PBO (pg/ml) mean ± SE
Control subjects pool*	< 1.9
Patient sample at diagnosis during anti-lice product exposure	48.3 ± 2.0
Patient sample during anti-lice product exposure and treatment with cortisone acetate	44.8 ± 1.8
Patient sample 6 months after anti-lice product discontinuation	< 1.9

SE = standard error; *Controls sample was represented by a pool of female serum leftover samples from routine analyses

### Evaluation of PBO effects on cell number

PBO effects on GloHEK cell viability were assessed to avoid toxic concentrations. We therefore evaluated GloHEK cell number after 48 and 72 h of incubation without and with PBO, Dex and Mif at increasing concentrations (up to 100 mM). We then calculated EC_50_ of each compound on viable cell number, as shown in Table 4. These results indicate that the tested compounds do not affect cell number at the concentration employed in the luciferase assay (10 µM).

**Table 4:** EC_50_ of PBO, Dex and Mif after 48 and 72 h on cell viability

Compounds	48 h	72 h
PBO	5 mM	1.8 mM
Dex	1.6 mM	0.70 mM
Mif	6 mM	1.9 mM

### Evaluation of PBO glucocorticoid-like activity

**Fig. 1:**
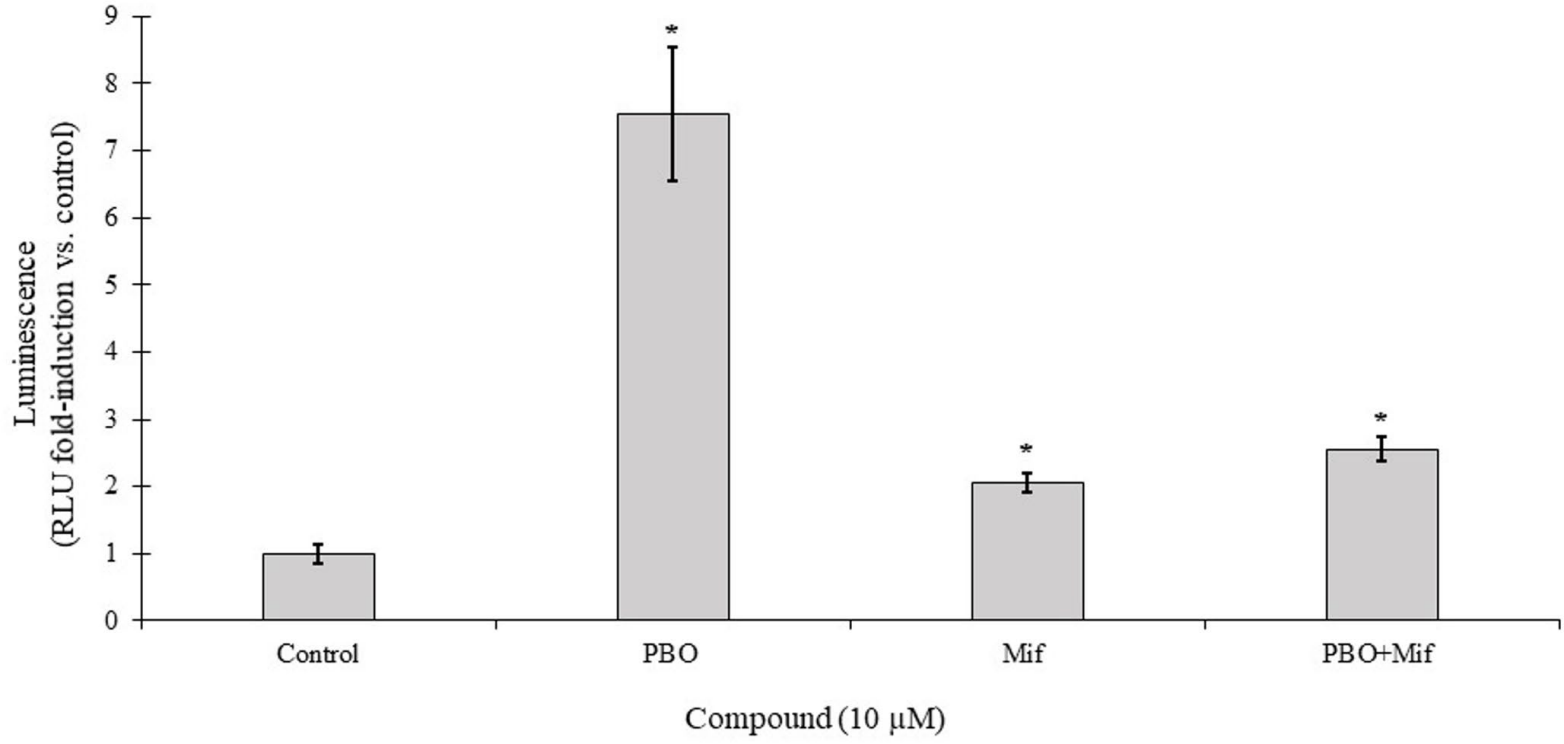
GC-like activity evaluation of PBO. GloHEK cells were incubated in 96-well plates for 24 h in a culture medium supplemented with 10 µM PBO and 10 µM Mif alone or in combination. Control cells were treated with vehicle solution. Luciferase activity was evaluated as described in the Materials and Methods section in 7 independent experiments in 3 replicates for control samples and each compound. Luminescence is expressed as percent mean RLU ± SE vs. control. *p < 0.001 vs. control

To verify a possible GC-like activity of PBO, we evaluated LUM in GloHEK cells incubated with 10 µM PBO, Dex and Mif alone or in combination for 24 h, as indicated by the assay manufacturer. We found that Dex significantly (*p* < 0.05) induced LUM (52.0 ± 3.5 RLU) as compared to control untreated cells (20.6 ± 1.7 RLU), in line with the manufacturer’s indications, confirming that our experimental model was useful to assess GC-like activity of investigational drugs. As shown in Fig. 1, PBO significantly increased baseline LUM by > 7-fold (*p* < 0.001).

On the other hand, Mif enhanced baseline LUM by ~ 2-fold (*p* < 0.001) and potently hampered PBO-induced LUM (*p* < 0.01). These results support the hypothesis that PBO has a GC-like effect.

## Discussion

CS has an estimated variable incidence of 2–5 new cases per million people each year and is defined as a rare disease. It is even less frequent in pediatric subjects, who make up 10% of all CS diagnoses. In children, the most frequent CS cause is iatrogenic, due to the use of GC to treat respiratory, dermatological, autoimmune, neoplastic conditions [9]. However, the CS case described here is unusual, as it results from inappropriate chronic exposure to an apparently harmless product, an anti-lice foam. CS diagnosis in children is often delayed by 2.5 ± 1.7 years [10], mainly attributable to the rarity of this clinical condition and mild phenotypic signs and symptoms in the early stages of the disease. The most frequent clinical sign is weight gain (98% of cases), which becomes particularly indicative when associated with growth retardation. Other typical CS clinical signs include hirsutism and oligo-amenorrhea in post-pubertal age, while symptoms such as fatigue, striae rubrae and acne are found in 61%, 49% and 44% of pediatric patients, respectively [11]. In the described case, the association of symptoms such as hirsutism, acne, oligomenorrhea, striae rubrae and weight gain suggested a possible CS, even in the presence of normal height. Low ACTH and cortisol plasma levels (the latter both basal and after ACTH stimulation) indicated a picture of adrenal insufficiency. Therefore, we tried to rule out all possible causes of exogenous GC exposure. Since medications and other products were excluded, we hypothesized that the clinical picture was associated with chronic exposure to the anti-lice product. The noteworthy normalization of the patient’s hormone levels and the striking improvement in CS signs and symptoms six months after anti-lice foam discontinuation, supported the hypothesis of an exogenous GC exposure. In addition, the clinical course showed a complete functional recovery of the hypothalamic-pituitary-adrenal (HPA) axis, differently from previously reported cases [5, 12], suggesting that the GC-like activity of the anti-lice product is completely reversible. Two components were found to be of interest in the foam formulation: pyrethrins and PBO. We were unable to measure circulating pyrethrins possibly due to their short half-life (~ 12 h), high photosensitivity and poor absorption through the skin [13]. Pyrethrin formulations usually contain PBO, which hampers their metabolic degradation. Previous evidence showed that PBO absorption after skin exposure for short time intervals (from 8 h to 7 days) is very low [14, 15], but data on longer exposure times are lacking. Our data show that elevated PBO serum levels are found after prolonged exposure (one year) to products containing this compound. Our results support the hypothesis that PBO has a specific GC-like activity. The LUM measured from transfected GloHEK cells following PBO exposure reflects PBO-induced GR transcriptional activity, which was antagonized by Mif. The evidence that PBO may have a transcriptional activity is also provided by the demonstration that it induced liver cytochrome P450 (CYP) 1A2 in a murine model [16]. Similarly, PBO elicited a robust CYP transcriptional response in Drosophila melanogaster. This effect was both time-and concentration-dependent, suggesting that the degree of P450 activity inhibition correlates with the magnitude of the transcriptional response [17].

Therefore, CS signs and symptoms in our patient may be due to the inappropriate prolonged exposure to the anti-lice compound, causing a significant increase in PBO serum levels over time. On the other hand, shorter exposure is unlikely to cause similar effects [14, 15]. The reported PBO tissue-specific effects in the Drosophila model [17] may explain the peculiar clinical picture of our patient, who displayed some but not all canonical CS signs and symptoms. In addition, our patient showed HPA axis suppression with very low plasma cortisol levels. PBO induction of liver CYP activity may contribute, at least in part, to this clinical picture, but evidence in humans is lacking. Overall, clinical and experimental data indicate that PBO has a GC-like activity. Okajima and Hertting showed that PBO hampers ACTH response to AVP in murine pituitary cells, possibly via CYP involvement [18]. The GC-like activity of PBO, as described in our study, may indeed explain their findings. Impaired ACTH response to AVP might be due to negative feedback exerted by PBO on pituitary cells. However, we cannot completely rule out possible alternative mechanisms (e.g., upstream signalling, indirect modulation).

The inappropriately prolonged and intensified use of the anti-lice product by the patient is certainly one of the distinctive aspects of this exogenous CS pediatric case associated with a possible individual susceptibility to GC. It is important to emphasize that the patient fully recovered after anti-lice product discontinuation.

This study presents several limitations. First, the evidence is derived from a single case, which limits the generalizability of our findings and prevents definitive causal inferences. Moreover, serum PBO levels were compared with those from a pool of unselected controls, which may not fully reflect age-matched background exposure. Pyrethrins were not evaluated in vitro due to the fact that we did not find them in the patient’s serum and to their short half-life. Finally, we cannot exclude other unrecognized or unmeasured environmental exposures that might have contributed to hypothalamic-pituitary-adrenal axis suppression or modified the patient’s susceptibility to PBO. Despite these limitations, this case prompts awareness among clinicians, caregivers, and regulatory bodies towards “natural products”.

In conclusion, PBO reached elevated serum concentrations during chronic anti-lice product use and declined after its discontinuation. In a GR reporter assay, PBO activated GR-dependent transcription at 10 µM, and the effect was antagonized by mifepristone. While these findings are consistent with GR-mediated activity, additional studies are needed to define direct binding and in-vivo relevance.

This case report underlines the importance of considering exogenous causes of CS [19], especially in pediatric patients, where everyday products may also cause the syndrome. Therefore, an accurate medical history, including detailed information on the use of non-pharmacological products, is essential for a complete and, above all, rapid diagnostic process. Moreover, this case report highlights the importance of following the instructions for the use of dermatological or antiparasitic treatments, such as anti-lice products. Knowledge concerning product application may avoid foam Cushing.

## Data Availability

Data available on reasonable request.
